# System Error Compensation Methodology Based on a Neural Network for a Micromachined Inertial Measurement Unit

**DOI:** 10.3390/s16020175

**Published:** 2016-01-29

**Authors:** Shi Qiang Liu, Rong Zhu

**Affiliations:** State Key Laboratory of Precision Measurement Technology and Instrument, Department of Precision Instruments, Tsinghua University, Beijing 100084, China; liusq13@mails.tsinghua.edu.cn

**Keywords:** micromachined inertial measurement unit, inertial sensor, thermal gas sensor, error compensation, neural network

## Abstract

Errors compensation of micromachined-inertial-measurement-units (MIMU) is essential in practical applications. This paper presents a new compensation method using a neural-network-based identification for MIMU, which capably solves the universal problems of cross-coupling, misalignment, eccentricity, and other deterministic errors existing in a three-dimensional integrated system. Using a neural network to model a complex multivariate and nonlinear coupling system, the errors could be readily compensated through a comprehensive calibration. In this paper, we also present a thermal-gas MIMU based on thermal expansion, which measures three-axis angular rates and three-axis accelerations using only three thermal-gas inertial sensors, each of which capably measures one-axis angular rate and one-axis acceleration simultaneously in one chip. The developed MIMU (100 × 100 × 100 mm^3^) possesses the advantages of simple structure, high shock resistance, and large measuring ranges (three-axes angular rates of ±4000°/s and three-axes accelerations of ±10 g) compared with conventional MIMU, due to using gas medium instead of mechanical proof mass as the key moving and sensing elements. However, the gas MIMU suffers from cross-coupling effects, which corrupt the system accuracy. The proposed compensation method is, therefore, applied to compensate the system errors of the MIMU. Experiments validate the effectiveness of the compensation, and the measurement errors of three-axis angular rates and three-axis accelerations are reduced to less than 1% and 3% of uncompensated errors in the rotation range of ±600°/s and the acceleration range of ±1 g, respectively.

## 1. Introduction

With the advancements of micromachined accelerometers and gyroscopes, miniaturized inertial measurement unit (MIMU) has attracted more and more attentions in recent years due to its small size, batch fabrication, low cost, and low power consumption. Nowadays, MIMU plays an important role in civil and military applications (e.g., smartphones, wearable equipment, motion tracking equipment, vehicle navigation, *etc.*) [1,2,3,4]. In general, conventional MIMU with six degrees of freedom is a three-dimensional integrated system consisting of three uniaxial micromachined accelerometers (or a three-axis accelerometer) and three uniaxial micromachined gyroscopes (or a three-axis gyroscope) mounted orthogonally in a Cartesian coordinate system. The accelerometers and gyroscopes measure the accelerations and angular rates around corresponding three-axes, respectively. Theoretically, each output of the inertial sensors is proportional to the angular rate or acceleration around the corresponding axis of the system and, thus, three orthogonal angular rates and three orthogonal accelerations can be measured directly from the sensor outputs in MIMU. As a matter of fact, however, the outputs of the inertial sensors may be coupled with each other, containing deterministic errors and stochastic errors, all of which corrupt the system accuracy [5]. Stochastic errors are usually represented as bias instability and random walk which can be restrained with filtering technologies, such as prediction error minimization (PEM) method, autoregressive (AR) processes, wavelet multi-resolution techniques, and so on [6,7]. Deterministic errors in a MIMU system are mainly induced by cross-coupling, misalignment, and eccentric problems when assembling multiple sensors into a three-dimensional system, and it has been experimentally demonstrated that they result in navigation errors with quadratic or cubic growth in time [8]. Compensation, often termed MIMU calibration, is generally used to eliminate the deterministic errors by using error-model-based calibration. Presently, many methods of MIMU calibration have been reported, such as multi-position static calibration [9,10,11], fast field calibration [12], and optical alignment calibration [13]. These methods usually need to establish a physical error model prior to the calibration process through an analytical method. For example, the model of misalignment error can be formulated by a direction cosine matrix [13]. However, some errors, such as cross-coupling, are generally difficult to be physically modeled due to their complexity, multivariability, coupling, and nonlinearity. Therefore, an effective system identification method needs to be investigated to solve this problem and, consequently, eliminate the system errors to the greatest extent.

Conventional micromachined inertial sensors, such as vibrating shell gyroscopes [14], tuning-forks gyroscopes [15], and vibrating beams gyroscopes [16], need a mechanical proof mass suspended on springs, which raises complexities of structure and fabrication, and also restricts the high-shock resistance of the device [1]. Another kind are fluidic inertial sensors, which use a fluidic medium instead of mechanical proof mass as the key moving and sensing elements. According to the driving principle of the fluidic medium, the sensor can be categorized into the jet flow gyroscope based on jet flow [17,18], the thermal accelerometer, and gyroscope based on thermal convection [19,20,21,22]. Another gas inertial sensor based on thermal expansion was recently reported and demonstrated simple structure, high shock resistance, and wide measuring range [23], which is able to measure one-axis angular rate and one-axis acceleration simultaneously in one chip. However, the thermal inertial sensors exhibit obvious cross-coupling errors among axes and between rotation and acceleration measurements because of the complex thermal fluid motion inside the sensor chamber [23,24], such as the influence of the gravitational acceleration on the gyro output at a vertical position of the sensor.

In this paper, we present a thermal gas MIMU system using only three thermal gas inertial sensors to realize measurements of three-axis angular rates in the range of ±4000°/s and three-axis accelerations in the range of ±10 g. To overcome the complex cross-coupling errors and other system errors of the thermal gas MIMU, we propose a compensation method using a three-layer back propagation (BP) neural network to model the complex and nonlinear error system for eliminating system errors of a MIMU. The parameters of the neural network are estimated by using experiment-based identification. Experiments validate the effectiveness of the compensation. Results also indicate that the neural network-based compensation method is effective for eliminating not only the cross-coupling error but also the errors induced from other various sources, such as misalignment and eccentricity. Compared with other compensation methods, the proposed compensation method is easily operated, physical-model-free, universal, and with good feasibility.

## 2. Design of Thermal Gas MIMU

### 2.1. Thermal Gas Inertial Sensor Used in the Gas MIMU

**Figure 1:**
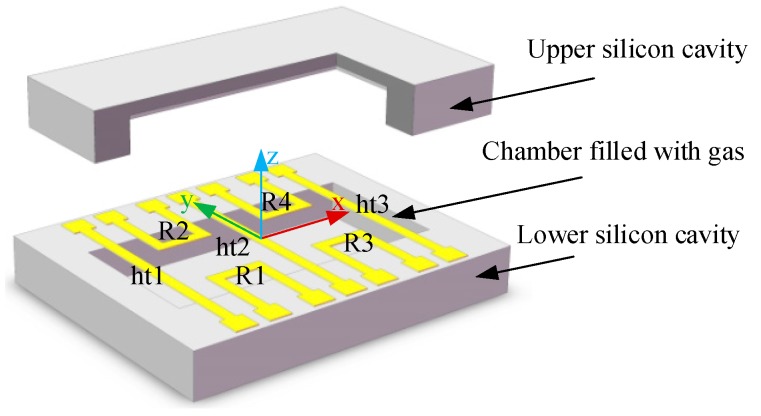
Schematic diagram of the thermal gas inertial sensor.

**Figure 2:**
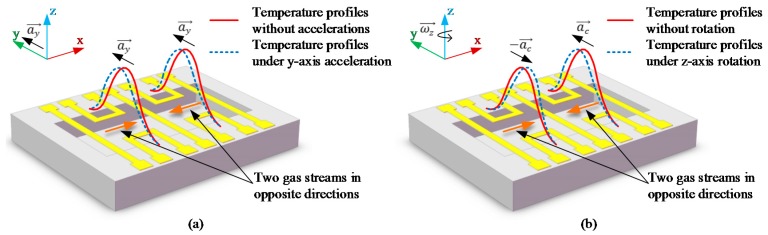
Temperature distribution deflected by a linear acceleration (**a**) and Coriolis acceleration (**b**).

The core components of the developed thermal gas MIMU are the micromachined thermal gas inertial sensors based on thermal expansion shown in Figure 1 and Figure 2 [23,25]. A sensor’s body coordinate frame *x*-*y*-*z* is defined, where the x-y plane is the working plane of the sensor and the z-axis is perpendicular to the working plane. The configuration of the thermal gas inertial sensor comprises three heater wires (ht1, ht2, ht3) and four thermistor wires (R1, R2, R3, R4) which are distributed in the working plane and suspended over a sealed micro chamber filled with a gas medium. By heating and cooling the central heater (ht2) and the two side heaters (ht1 and ht3) alternately, two seismic gas streams in opposite directions along the x-axis are generated in the working plane and the temperature profiles illustrated as red solid lines in Figure 2 are formed in the chamber. The *y*-axis linear acceleration deflects the temperature profiles on the two sides of the *y*-axis in the same direction along *y*-axis (shown in Figure 2a), while the rotation around the *z*-axis deflects the temperature profiles on the two sides of the *y*-axis in the opposite direction along the *y*-axis due to the opposite Coriolis accelerations on the two sides (shown in Figure 2b). These deflections of the temperature profile induced by either *y*-axis linear acceleration or *z*-axis rotation are detected by using the distributed four thermistors, outputs of which are used to deduce the y-axis acceleration and *z*-axis angular rate [23]. Therefore, the thermal gas inertial sensor has an inherent ability to measure the *y*-axis acceleration and the *z*-axis rotation simultaneously in one chip. The structure of the sensor has been optimized in our previous work [23] to restrain thermal convection and enhance thermal expansion, resulting in merits of simple structure, high shock resistance, large-range rotation gauge, and low cross-coupling effects.

### 2.2. Design of the Thermal Gas MIMU

**Figure 3:**
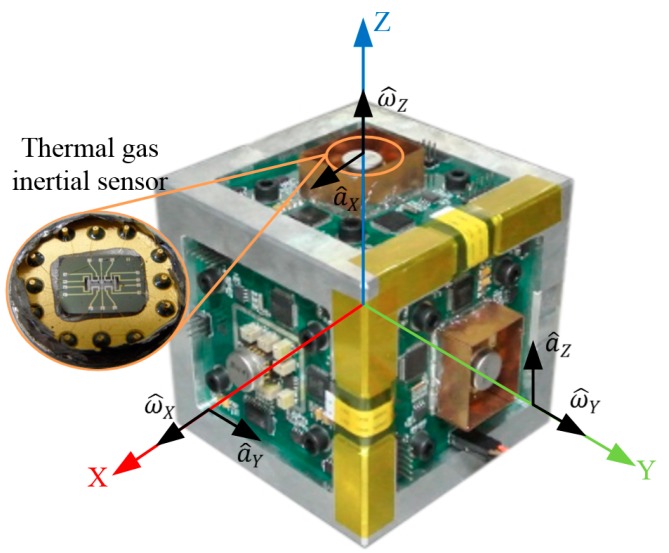
Configuration of the thermal gas MIMU.

In this paper three thermal gas inertial sensors are integrated together to construct a MIMU. The developed prototype of the MIMU is shown in Figure 3. Each sensor is mounted on a PCB, where the conditioning circuit of the sensor is located. The three PCBs are assembled orthogonally to form a three-dimensional frame. A rectangular coordinate frame *X*-*Y*-*Z* is defined as the MIMU’s body frame, where *X*, *Y*, and *Z* axes are served as sensing axes of the MIMU. On each axis of *X*-*Y*-*Z*, there is located a thermal gas inertial sensor, the outputs of which correspond to one-axis acceleration and one-axis angular rate. Specifically, the outputs of the sensor on the *X*-axis (${\hat{a}}_{Y}$,${\hat{\omega }}_{X}$) corresponds to *Y*-axis acceleration $a_{Y}$ and *X*-axis angular rate $\omega_{X}$; that of the sensor on the *Y*-axis (${\hat{a}}_{Z}$,${\hat{\omega }}_{Y}$) corresponds to *Z*-axis acceleration $a_{Z}$ and Y-axis angular rate $\omega_{Y}$ and that of the sensor on the *Z*-axis (${\hat{a}}_{X}$,${\hat{\omega }}_{Z}$) corresponds to *X*-axis acceleration $a_{X}$ and *Z*-axis angular rate $\omega_{Z}$.

**Figure 4:**
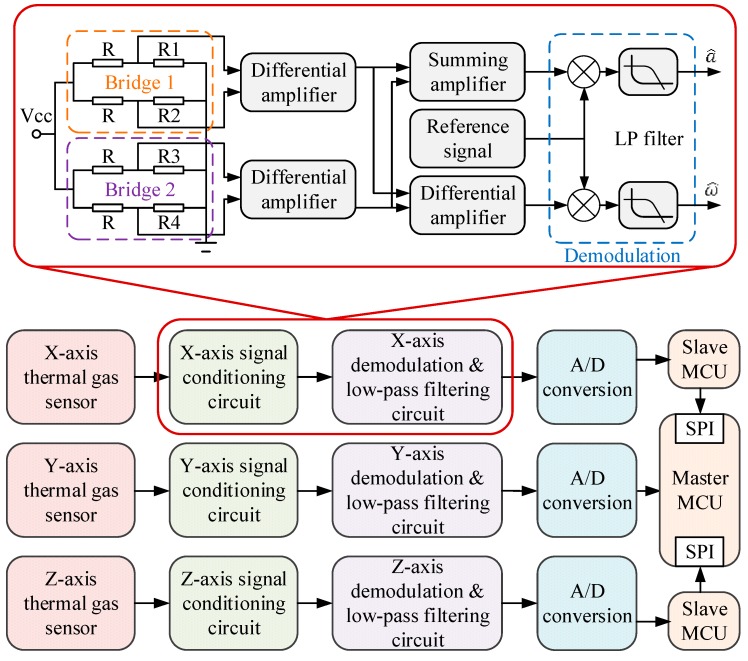
Schematic diagram of conditioning circuit of the thermal gas MIMU.

A diagram of the measurement and control circuit of the MIMU is illustrated in Figure 4, where the conditioning circuit of the thermal gas inertial sensor is also demonstrated. The sensor circuit consisting of two Wheatstone bridges, two-level differential amplifying circuit, demodulation circuit, and low-pass filtering circuit are used to implement the conversions from the deflections of the temperature profile to the quantities of acceleration and rotation. Each sensor has one 16-bit A/D converter and one micro control unit (MCU) to extract one-axis acceleration and one-axis angular rate which makes each circuit board independent and avoids crosstalk. A high-performance MCU acts as a master for operating compensation algorithm.

As mentioned above, each thermal gas inertial sensor is capable of simultaneously measuring one-axis acceleration and one-axis rotation. Three gas inertial sensors are perfectly adequate to measure three-axis accelerations and three-axis rotations, which simplifies the MIMU structure and operating circuit, miniaturizes the system size, and reduces the power consumption and fabrication cost of the MIMU.

## 3. Error Source Analysis of the MIMU

In general, error sources of a MIMU mainly consist of cross-coupling, misalignment, eccentricity, noise, *etc.* Among them, the cross-coupling error is significant for a thermal gas MIMU due to the complex fluidic motion in the sensor chamber. The cross-coupling effects include the coupling errors among the measurements of three-axis accelerations and three-axis angular rates. Although an impressive decrease of the coupling was obtained via using thermal expansion instead of thermal convection [23], the cross-coupling error still cannot be ignored for the gas MIMU in practical applications.

According to our previous studies [23,24], there are two types of fluid motions in the gas inertial sensors, thermal convection flow and thermal expansion flow. The fluid motion driven by thermal convection is dependent on the acceleration and, thus, results in cross-coupling effects between acceleration and rotation measurements of the sensor, while the fluid motion driven by thermal expansion is independent of the acceleration. A non-dimensional momentum model characterizing the kinetic process of the three-dimensional flow in a chamber of a thermal gas sensor is formulated as [23]:

(1) $$\frac{\partial \bar{\vec{U}}}{\partial \delta }+\bar{\vec{U}}\cdot \bar{\nabla }\bar{\vec{U}}+\bar{\vec{U}}\left(\bar{\nabla }\cdot \bar{\vec{U}}\right)=\vec{Gr}\Delta \Theta +\alpha_{v}\frac{\partial T}{\partial \delta }\bar{\vec{U}}-2\bar{\vec{\Omega }}\times \bar{\vec{U}}-\bar{\vec{\Omega }}\times \left(\bar{\vec{\Omega }}\times \bar{\vec{r}}\right)+\bar{\nabla }\cdot \bar{\tau }$$

where the Grashof number $\vec{Gr}=\left(Gr_{x},Gr_{y},Gr_{z}\right)=\left(\frac{\alpha_{v}\left(T_{h}-T_{0}\right)a_{x}H^{3}}{v^{2}},\frac{\alpha_{v}\left(T_{h}-T_{0}\right)a_{y}H^{3}}{v^{2}},\frac{\alpha_{v}\left(T_{h}-T_{0}\right)a_{z}H^{3}}{v^{2}}\right)$; $\bar{\nabla }={\left(\frac{\partial }{\partial \bar{x}},\frac{\partial }{\partial \bar{y}},\frac{\partial }{\partial \bar{z}}\right)}^{T},\bar{\tau }=\bar{\nabla }\bar{\vec{U}}+{\left(\bar{\nabla }\bar{\vec{U}}\right)}^{T}-\frac{2}{3}\sigma \bar{\nabla }\cdot \bar{\vec{U}}$; $\bar{\vec{U}}=\frac{\vec{U}}{\left(\frac{v}{H}\right)}={\left(\frac{U_{x}}{\frac{v}{H}},\frac{U_{y}}{\frac{v}{H}},\frac{U_{z}}{\frac{v}{H}}\right)}^{T},\bar{\vec{r}}=\frac{\vec{r}}{H}={\left(\frac{x}{H},\frac{y}{H},\frac{z}{H}\right)}^{T},$ $\Theta =\frac{T-T_{0}}{T_{h}-T_{0}},\bar{\vec{\Omega }}=\frac{\vec{\Omega }}{\left(\upsilon /H^{2}\right)}={\left(\frac{\Omega_{x}}{\upsilon /H^{2}},\frac{\Omega_{y}}{\upsilon /H^{2}},\frac{\Omega_{z}}{\upsilon /H^{2}}\right)}^{T}$; $\delta =t\upsilon /H^{2}$; $T_{h}$ is the heater temperature; $T_{0}$ refers to the ambient temperature; $\vec{r}=\left(x,y,z\right)$ refers to the position vector in the sensor’s body coordinate frame; angular rate vector $\vec{\Omega }=\left(\Omega_{x},\Omega_{y},\Omega_{z}\right)$; $\vec{U}={\left(U_{x},U_{y},U_{z}\right)}^{T}$ refers to fluid velocity vector $\vec{f}={\left(a_{x},a_{y},a_{z}\right)}^{T}$ refers to the acceleration vector; υ($\upsilon =\frac{\mu }{\rho }$) is momentum viscosity; H is the feature dimension; the coefficient of thermal expansion $\alpha_{v}=\frac{1}{V}\frac{\partial V}{\partial T}{|}_{p}=-\frac{1}{\rho }\frac{\partial \rho }{\partial T}{|}_{p}\approx -\frac{1}{\rho }\frac{\rho -\rho_{0}}{T-T_{0}}$; $\nabla P=\rho_{0}\vec{f}$; $\rho_{0}$ refers to the gas density at the ambient temperature; ρ, P, μ, T are gas density, pressure, dynamic viscosity, and temperature in the sensor chamber.

In Equation (1), the item of $\vec{Gr}\Delta \Theta$ is a driving force to generate a thermal convection flow, while the term of $\alpha_{v}\frac{\partial T}{\partial \delta }\bar{\vec{U}}$ is a driving force to generate the thermal expansion flow. Obviously, the former is correlative with the Grashof number $\vec{Gr}$, which is dependent on the acceleration $\vec{f}={\left(a_{x},a_{y},a_{z}\right)}^{T}$, while the latter is correlative with the transient variation rate of the temperature that is independent of the acceleration. From Equation (1), it is theoretically demonstrated that the thermal convection flow is generally existent in a fluid dynamic system, which unavoidably results in a cross-coupling between acceleration and rotation measurements, such as the influence of the gravitational acceleration on the gyro output at a vertical position of the sensor. In addition, the problems are more complex due to misalignment and asymmetry of the sensor structure induced in fabrication, thermal distortion in the operation of the sensor, all of which will induce the cross-coupling effects. Consequently, the actual couplings exhibit a complex interactive relationship between not only rotation and acceleration measurements, but also rotation measurements among axes and acceleration measurements among axes. In addition, it is seen that other inertial sensors based on thermal principles all suffer cross-coupling effects, such as thermal jet gyroscopes and thermal accelerometers.

In addition to the cross-coupling errors, misalignment is another error source of the MIMU. Misalignment errors generally exist when assembling multiple sensors into a three-dimensional system, which bring about negative effects on the system accuracy. In addition, assembly positions of individual sensor deviated from the centroid of the MIMU system will also result in assembling errors (termed as eccentric errors) in the system. In addition of above deterministic errors, there are stochastic errors, which are not a concern of this paper.

## 4. Error Compensation Method Based on a BP Neural Network

### 4.1. System Error Modelling

The basic idea of the proposed compensation method is to establish a relationship model between the outputs of the sensors and the physical quantities to be measured. For a MIMU, the quantities to be measured are the three-axis accelerations and three-axis angular rates along the *X*-*Y*-*Z* axes denoted as $a=\left(a_{X},\;a_{Y},\;a_{Z}\right)$ and $\omega =\left(\omega_{X},\;\omega_{Y},\;\omega_{Z}\right)$, respectively. The outputs of the sensors in the MIMU are the voltage outputs of the sensors representing three-axis acceleration outputs and three-axis angular rate outputs denoted as $\;\hat{a}=\left({\hat{a}}_{X},\;{\hat{a}}_{Y},\;{\hat{a}}_{Z}\right)$ and $\hat{\omega }=\left({\hat{\omega }}_{X},\;{\hat{\omega }}_{Y},\;{\hat{\omega }}_{Z}\right)$, respectively. Obviously, the relationship model of the MIMU is a multi-input and multi-output (MIMO) coupling system, which can be formulated by:

(2) $$\left[a,\;\omega \right]=f\left(\left[\hat{a},\hat{\omega }\right]\right)$$

In general, the work for error compensation focuses on mapping relationship f which is a multivariate and nonlinear function. Analytical methods are commonly used for approximating f, but are usually too complicated and inefficient, especially for cross-coupling effects as mentioned above. An alternative method is experimental identification, which is more reliable and effective. In this paper, a system identification method based on experiment to model f is proposed using a three-layer back propagation (BP) neural network as the model structure. A BP neural network is simpler and more mature than other neural networks. Moreover, it has been theoretically proven that three layers of a neural network could solve arbitrarily complicated nonlinear mapping problems [26].

**Figure 5:**
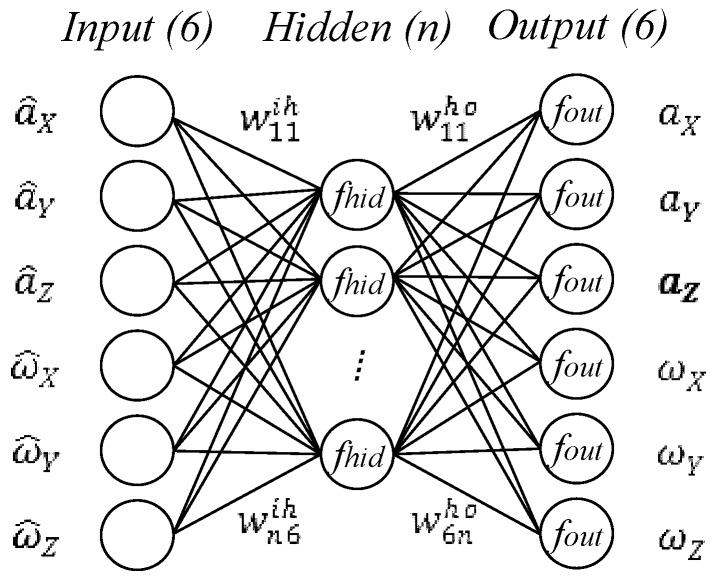
Structure of three-layer back propagation (BP) neural network to model the errors of the MIMU.

Illustrated in Figure 5, the structure of the BP neural network with six inputs $\left[\hat{a},\hat{\omega }\right]$ and six outputs [a, ω] was used to represent the model of f. The network includes several hidden neurons, the number of which was determined by trial. In Figure 5, the transfer function of the hidden layer *f_hid_*, the function of output layer *f_out_*, and transfer matrix ***w^ih^*** and ***w^ho^*** are the key parameters of the network model structure. These parameters, including the transfer functions *f_hid_* and *f_out_*, the number of hidden neurons, transfer weights ***w^ih^*** and ***w^ho^*** need to be determined through training using sufficient experimental data, in which the outputs of the gas inertial sensors were used as the inputs of the network, while the corresponding data of the actual accelerations and angular rates were used as the outputs of the network. The training of the neural network is to search for appropriate network parameters to minimize the sum-squared error between the network outputs and actual values by using an iteration algorithm. In the standard back propagation learning algorithm [26], two key parameters, including the learning rate and momentum parameter, need to be configured properly. The learning rate determines the increments of the transfer weights in each iteration step. A large learning rate leads to a fast learning but may result in non-convergence. The additional momentum makes it possible to avoid dropping into local minimum, which improves the efficiency of convergence in training. The BP learning algorithm with a learning rate of 0.005 and a momentum parameter of 0.5 is used in our training. The transfer functions *f_hid_* and *f_out_* play important roles in the model structure and accuracy. The hidden layer *f_hid_* usually uses tan-sigmoid or log-sigmoid function and the output layer *f_out_* often uses log-sigmoid or pure-line functions. Through experimental trials, we finally select a tan-sigmoid function of *f_hid_* and a pure-line function of *f_out_* according to the accuracy of the model. The number of hidden neurons is another key parameter dominating system accuracy and structural simplicity. A small number of hidden neurons will cause large fitting errors because there are not enough to model the MIMU system accurately, while too many hidden neurons will be redundant, which require more calculations but do not help to improve the model accuracy. Considering both accuracy and simplicity, the number of hidden neurons is finally determined to be 120 through trials that gradually increase the number of hidden neurons from a small number until the decrease of the sum-squared error becomes steady. MATLAB is used to develop the neural network.

### 4.2. Calibration Method

Calibration is an experimental method to obtain a group of sample data used for training the above neural network model. The acquired data of the sample should be sufficient, by which the working conditions of the MIMU are represented.

**Figure 6:**
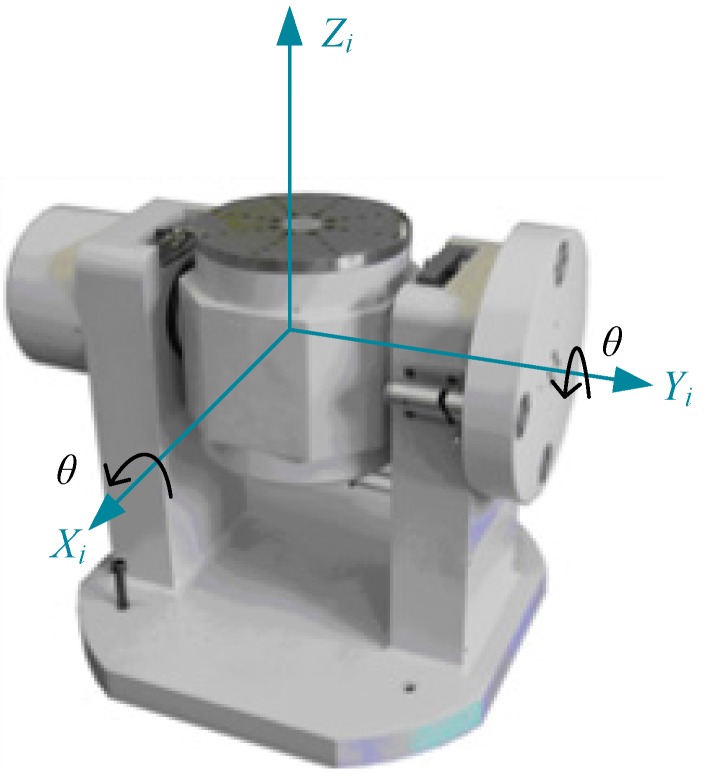
Two-axis turntable used for calibration.

The calibration experiment is conducted, including three tests: multi-position static tests, multi-position turntable rotation tests, and centrifugation tests. A two-axis turntable (902E-1, AVIC Beijing Precision Engineering Institute Aircraft Industry the measurement range of rotation is ±600°/s, the resolution of angular rate is 0.001°/s, the angle accuracy around inner-axis and outer-axis is ±5″ and ±8″ respectively, the perpendicularity between the rotation axes of both turntables is ±5″, the leveling accuracy of the rotation axes is ±5″) shown in Figure 6 is used in the experiment. The turntable contains an electric slip ring used for signal transmission between the MIMU and other instruments. A reference coordinate frame *X_i_*-*Y_i_*-*Z_i_* is defined as North-West-Upwards, where the axes of *X_i_* and *Y_i_* lie in a horizontal plane and the axis of *Z_i_* points to the opposite direction of gravity.

**Figure 7:**
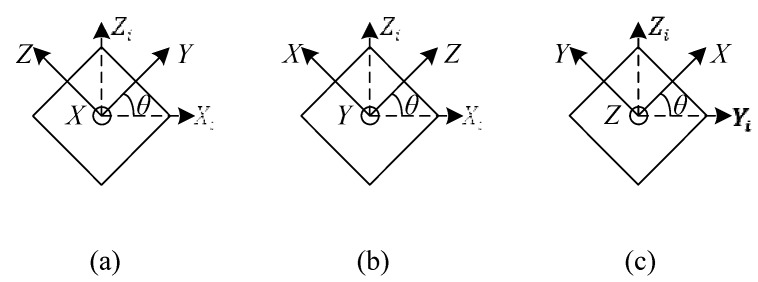
Processes of multi-position static calibration; (**a**) the rotation axis *X* is along *Y_i_*; (**b**) the rotation axis *Y* is along *Y_i_*; (**c**) the rotation axis *Z* is along *X_i_*.

The multi-position static tests are conducted to learn the coupling relationship between acceleration measurements among the *X*-*Y*-*Z* axes. The developed MIMU is mounted on the turntable and the *X*-*Y*-*Z* axes of the MIMU are adjusted, as shown in Figure 7, by rotating the turntable, where *θ* is a tilt angle which is applied from 0° to 360° at 5° per step. At each step, the outputs of the sensors and actual accelerations along the *X*-*Y*-*Z* axes are recorded. In this experiment, the actual accelerations to be measured are the gravity components along the *X*-*Y*-*Z* axes and the actual angular rates are approximately equal to zero without consideration of Earth rotation. The Earth rotation is much less than the noise level of the thermal gas inertial sensor (around 1°/s RMS [23]). Therefore, we ignored the Earth rotation in this study.

**Figure 8:**
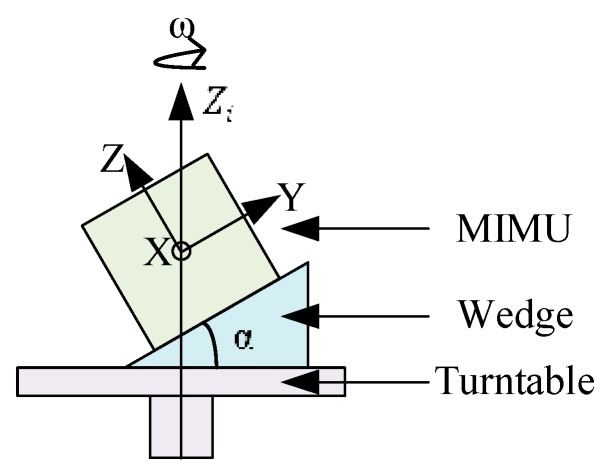
MIMU assembly scheme of multi-position turntable rotation rate calibration.

**Figure 9:**
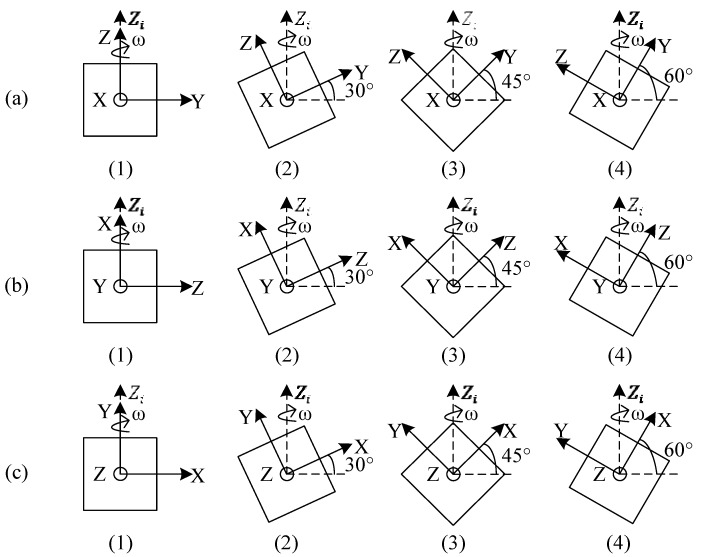
Processes of multi-position turntable rotation rate calibration; the tilt angle α is adjusted in the *Y*-*Z* plane (**a**) *X*-*Z* plane; (**b**) and *X*-*Y* plane; (**c**) respectively.

The multi-position turntable rotation tests are conducted to learn the coupling relationship between rotation measurements among the *X*-*Y*-*Z* axes as well as the coupling among rotation and acceleration measurements. Three cases of rotation with different tilt angles are conducted in this experiment. The rotation axis is set around $Z_{i}$-axis which is the opposite direction of gravity. As shown in Figure 8, the MIMU is mounted on a wedge (the angle accuracy is ±5″) with a tilt angle α and the wedge is mounted on the turntable to perform rotation tests, where α is set as 0°, 30°, 45°, and 60°, respectively, while the rotation axis is around the $Z_{i}$-axis. In the rotation test, the angular rate is applied from −600°/s up to 600°/s at 50°/s per step. At each step, the outputs of the sensors, actual angular rates, and accelerations along the *X*-*Y*-*Z* axes are recorded. The three cases are shown in Figure 9, where the *X*-*Y* plane, the *X*-*Z* plane, and the *X*-*Y* plane of the MIMU are adjusted, in turn, to be parallel with the slope of the wedge in cases (a), (b), and (c), respectively. In this experiment, the actual accelerations are the gravity components along the *X*-*Y*-*Z* axes and the actual angular rates are the rotation components along the *X*-*Y*-*Z* axes.

**Figure 10:**
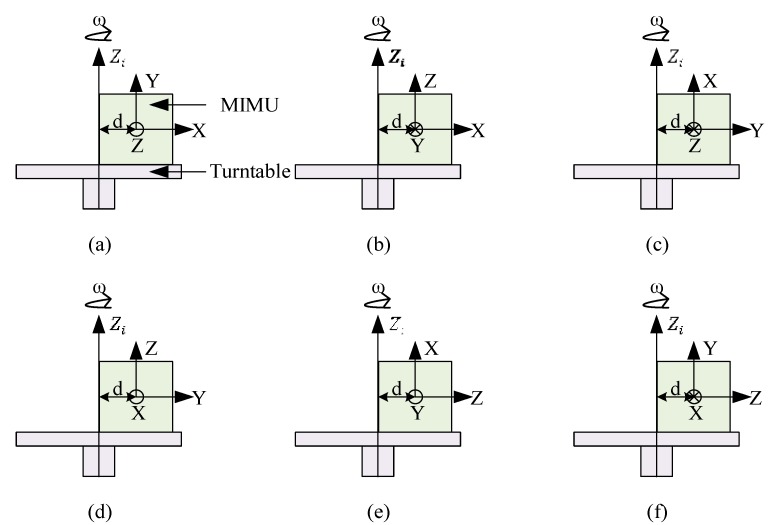
Processes of centrifugation calibration; the eccentric axis is set as the *X*-axis (**a**), (**b**), *Y*-axis (**c**), (**d**), and *Z*-axis (**e**), (**f**), respectively.

The centrifugation tests are conducted to learn more about the coupling relationship between acceleration and rotation measurements. Six cases of rotations with different eccentric distance *d* from the centroid of the MIMU are conducted in this experiment. The rotation axis is set around the $Z_{i}$-axis. As shown in Figure 10, the MIMU is mounted on the turntable with an eccentric distance *d* varying from −60 mm to 60 mm at an interval of 20 mm. At each eccentric position, the angular rate of the turntable around the $Z_{i}$-axis is applied from −600°/s to 600°/s at 50°/s per step, and at each step, the outputs of the sensors, actual angular rates and accelerations along the *X*-*Y*-*Z* axes are recorded. The eccentric axis is set as the *X*-axis, *Y*-axis, and *Z*-axis, respectively, in six cases as shown in Figure 10a–f. In this experiment, the actual accelerations are the gravity components combined with the centrifugal accelerations, and the actual angular rates are the angular rates around the vertical axis. Earth rotation is ignored in the experiment.

The sample data acquired in the calibration experiment are used to train the BP neural network to determine the parameters of the network, where the output voltages of the MIMU are used as inputs, and the corresponding data of the actual acceleration and angular rate are used as the outputs of the network.

## 5. Experimental Results and Discussions

**Figure 11:**
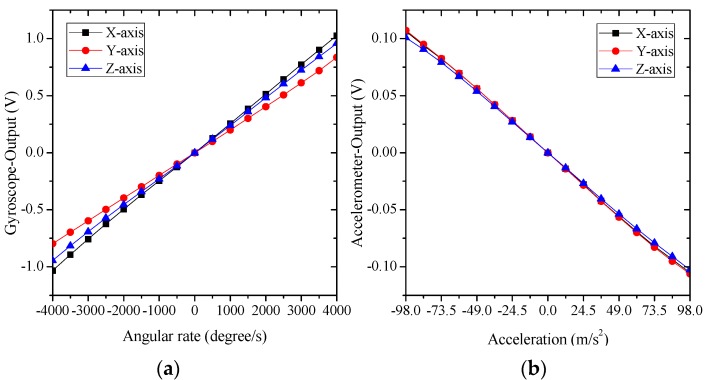
The experimental measurements of three-axis angular rates (±4000°/s) (**a**) and three-axis accelerations (±10 g); (**b**) using the MIMU.

The measurements of three-axis angular rates ranging from −4000°/s to +4000°/s and three-axis accelerations ranging from −10 g to +10 g are conducted for the developed thermal gas MIMU using an uniaxial turntable (920ET, AVIC Beijing Precision Engineering Institute Aircraft Industry, the measurement range of rotation is ±4000°/s; the resolution of angular rate is 0.001°/s). The acceleration measurements of ±10 g are conducted by using centrifugation of the turntable (the accuracy of the reference acceleration is about 60 ppm in the full measurement range). The experimental results shown in Figure 11 demonstrate a large measuring range of the gyro/rate sensor of the MIMU. The nonlinearity in full-scale range of rotation and acceleration is less than 2%.

**Figure 12:**
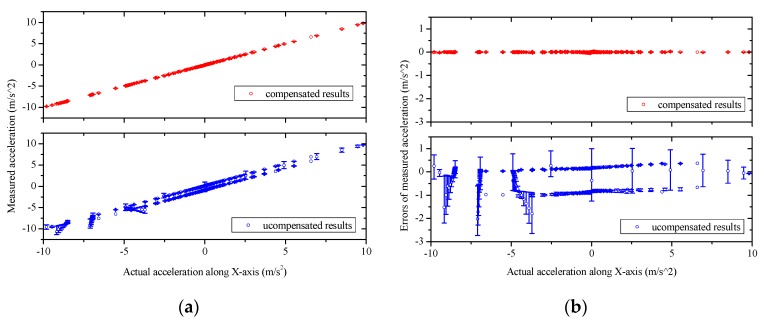

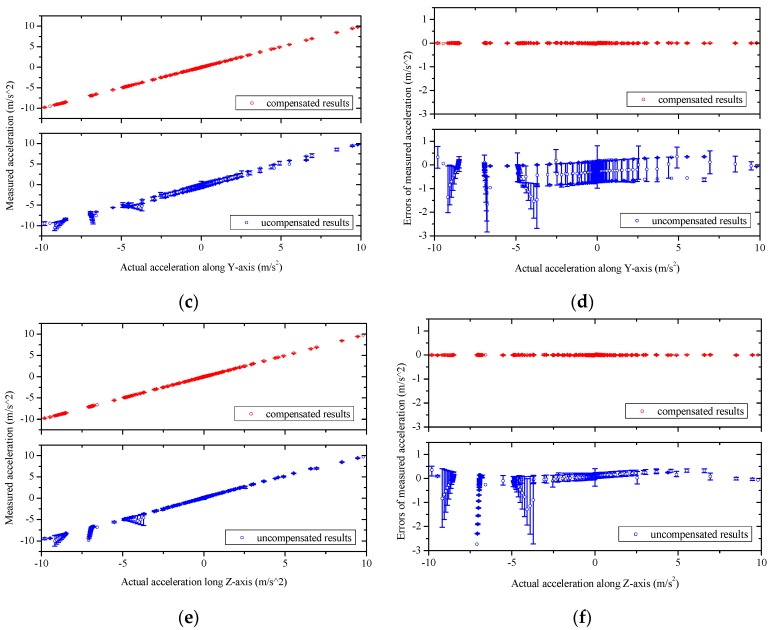
The acceleration measurements and the errors of the MIMU system with, and without, using the compensation method based on a neural network. (**a**), (**c**), and (**e**) refer to the measured acceleration along the *X*, *Y*, and *Z* axes, respectively, with and without compensation; (**b**), (**d**), and (**f**) refer to the measurement errors as functions of the actual accelerations along the *X*, *Y* and *Z* axes, respectively, with and without compensation. The asymmetric uncompensated errors are due to the amount of experiment data with negative values of the applied accelerations is more than that with positive values of the applied accelerations.

**Figure 13:**
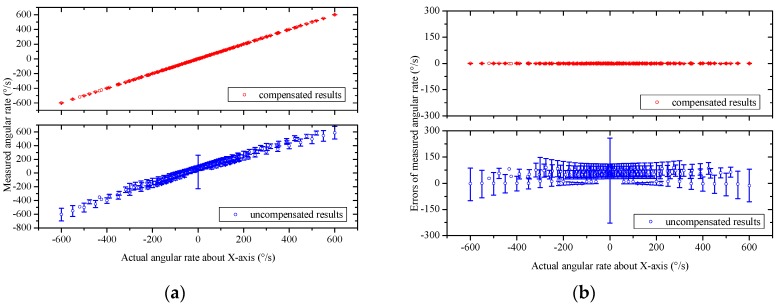

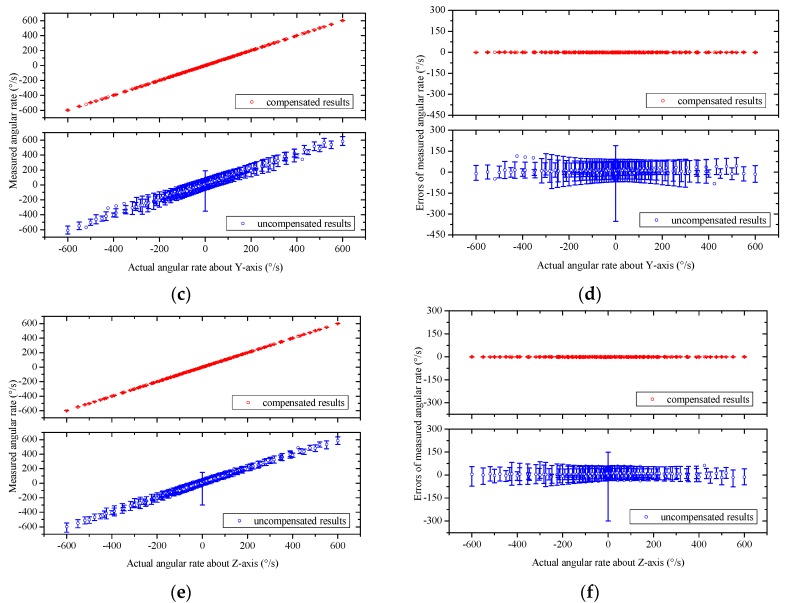
The angular rate measurements and the errors of the MIMU system with, and without, using the compensation method based on a neural network. (**a**), (**c**), and (**e**) refer to the measured angular rate around the *X*, *Y*, and *Z* axes, respectively, with and without compensation; (**b**), (**d**), and (**f**) refer to the measurement errors as functions of the actual angular rate around the *X*, *Y*, and *Z* axes, respectively, with and without compensation.

To test the cross-coupling performance of the MIMU, experimental calibrations and network training are conducted, as described in Section 4. A neural network model used for compensating system errors of the gas MIMU is therefore established. The established model is then employed in actual measurements to extract the accelerations and angular rates along the *X*-*Y*-*Z* axes using the sensor readouts of the gas MIMU. A validation experiment is further conducted to evaluate the compensation method following the similar processes of the calibration in Section 4 but with different rotations ranging from −600°/s to 600°/s and different accelerations ranging from −9.8 m/s^2^ to +9.8 m/s^2^. The output quantities of the MIMU compensated by using the neural network model (termed compensated results) are compared with the original output quantities of the MIMU without any compensation (termed uncompensated results) in the left figures of Figure 12 and Figure 13. The corresponding measurement errors are shown in the right figures of Figure 12 and Figure 13. Uncompensated results demonstrate significant errors, which are induced from coupling effects, misalignment, and assembling errors. These errors of the MIMU are greatly reduced by using the compensation method based on a neural network model.

The root mean square (RMS) errors of the gas MIMU system are listed in Table 1, which indicated that by using the neural network-based compensation method the measurement errors of three-axis angular rates and three-axis accelerations of the gas MIMU are reduced to less than 1% and 3% of the uncompensated errors in the rotation range of ±600°/s and the acceleration range of ±1 g, respectively. According to the component performances of the thermal gas inertial sensor [23], the angular rate noise of the sensor reaches around 1°/s RMS and the acceleration noise is around 1 mg RMS. The compensated errors of the developed MIMU have reached the noise level of the individual sensors, which implies the system errors are completely eliminated by using the compensation method.

**Table 1:** RMS errors of the measured accelerations and angular rates by the MIMU system with, and without, using network-based compensation method.

RMS Error	a*_X_* (m/s^2^)	a*_Y_* (m/s^2^)	a*_Z_* (m/s^2^)	ω*_X_* (°/s)	ω*_Y_* (°/s)	ω*_Z_* (°/s)
Compensated	0.0100	0.0085	0.0093	0.44	0.55	0.52
Uncompensated	0.5784	0.5129	0.3294	88.58	87.43	62.56
Comp./Uncomp. (%)	1.73	1.66	2.83	0.50	0.63	0.84

Conventional compensation methods are used to deal with specific error problem, such as nonlinear errors, misalignment errors of the MIMU. However, these existing methods are invalid to compensate complex and nonlinear cross-coupling errors exhibited in the proposed MIMU. It is the first time that a neural network is used to comprehensively compensate the system errors of the thermal gas MIMU, which are complex multivariate and nonlinear coupling, including cross-coupling errors, misalignment errors, eccentric errors, and any other deterministic errors. The proposed error compensation method is easily operated, physical-model-free and universal, which is applicable to not only compensate the system errors of the gas MIMU, but also overcome various deterministic errors in general integrated sensor systems.

## 6. Conclusions

A gas MIMU is developed by integrating only three thermal gas inertial sensors to measure three-axis accelerations (±10 g) and three-axis angular rates (±4000°/s). The errors of the gas MIMU are analyzed, which mainly contain cross-coupling errors, misalignment errors, and eccentric errors. A universal compensation method by using a neural network to model system errors is proposed to eliminate system errors of an integrated sensor system. The compensation method is applied to compensate the errors of the thermal gas MIMU. The experiments validate the effectiveness of the method and demonstrate that the measurement errors of three-axis angular rates and three-axis accelerations are reduced to less than 1% and 3% of uncompensated errors in the rotation range of ±600°/s and the acceleration range of ±1 g, respectively, by using the neural network-based compensation method, due to the limitation of our available experimental facility. It is evident that the compensation methodology can be easily scaled up to the full measurement range of the MIMU when using larger-range equipment.
